# Evaluation of GPT-5.2 for melanoma detection across skin tones

**DOI:** 10.3389/fmed.2026.1816102

**Published:** 2026-05-08

**Authors:** Katie L. Frederickson, Samuel E. Adunyah, Qingguo Wang

**Affiliations:** Department of Biochemistry, Cancer Biology, Neurosciences and Pharmacology, School of Medicine, Meharry Medical College, Nashville, TN, United States

**Keywords:** ChatGPT, dermoscopy, GPT-5.2, large language model, melanoma diagnosis, skintone

## Abstract

Malignant melanoma (MM) is the most aggressive form of skin cancer, for which early detection is critical and strongly associated with improved survival outcomes. Recent advances in large language models (LLMs), such as ChatGPT and Gemini, present promising opportunities to support melanoma early screening and clinical decision-making. However, despite increasing interest in LLM-based dermatologic applications, their diagnostic reliability across different populations remains insufficiently characterized. In this study, we systematically evaluated the performance of GPT-5.2 across skin pigmentation groups using Milk10K, a clinically curated, publicly available dermatology dataset comprising paired dermoscopic and clinical close-up images with histopathology-confirmed diagnoses and standardized skin tone annotations. GPT-5.2 was assessed on two clinically relevant tasks: binary malignancy discrimination and top-3 differential diagnosis. A balanced subset of 460 lesions (92 per skin tone class) was randomly selected for evaluation. Across both tasks and imaging conditions, GPT-5.2 showed moderate diagnostic performance, with broadly consistent accuracy, F1 score, and Cohen’s *κ* across skin tone groups, without evidence of systematic performance decline in darker skin tones. The incorporation of clinical close-up images provided modest improvements in overall performance while maintaining similar behavior across pigmentation classes. These findings suggest that GPT-5.2 exhibits stable melanoma-related diagnostic performance across diverse skin tones on this dataset. The study’s limitations and implications for future development are also discussed.

## 1 Introduction

Malignant melanoma (MM) is a malignancy of melanocytes, the pigment-producing cells of the skin. It is the most aggressive form of skin cancer and has a high propensity for metastasis to regional and distant organs. The American Cancer Society (ACS) projects approximately 112,000 new MM cases in 2026, including an estimated 65,400 cases in men and 46,600 in women (1). An estimated 8,510 deaths are expected in the same year.

Melanoma comprises several clinically distinct subtypes that differ in growth patterns, anatomical distribution, and diagnostic visibility. Superficial spreading melanoma (SSM) is the most common subtype and often exhibits the classic asymmetry, border irregularity, color variation, diameter, and evolution (ABCDE) features, whereas nodular melanoma (NM) frequently grows rapidly and may lack these characteristic signs, complicating early recognition. Acral lentiginous melanoma (ALM), which arises on the palms, soles, or nail apparatus, represents a relatively uncommon subtype in populations of European ancestry but constitutes a larger proportion of melanoma cases in individuals with darker skin tones. Melanoma *in situ* (MIS), the earliest stage of disease confined to the epidermis, offers the greatest opportunity for curative treatment when detected promptly. These heterogeneous clinical presentations contribute to diagnostic complexity and highlight the need for improved detection strategies across melanoma subtypes (2).

Early identification of melanoma is strongly associated with improved patient survival. However, diagnostic accuracy remains challenging because early melanoma lesions can resemble benign pigmented lesions such as nevi or seborrheic keratoses. These challenges may be further compounded by variations in skin pigmentation and lesion morphology across populations. Recent studies have therefore explored novel imaging and computational techniques to enhance melanoma detection. For example, Lin et al. have suggested that the spectrum-aided vision enhancer (SAVE) can improve the visualization of subtle pigmentary patterns and structural features in several melanoma subtypes, including ALM, MIS, NM, and SSM (3).

The timely detection of melanoma is constrained by limited access to dermatologic care in many areas. In the United States, dermatology residency programs offer only 574 positions annually, contributing to workforce constraints. A recent study reported a mean wait time of 50 days for a new patient dermatology appointment (4). These barriers to timely dermatologic evaluation may contribute to delayed detection, disease progression, and increased melanoma-related mortality rates.

The widespread adoption of large language models (LLMs) has the potential to help address persistent gaps in melanoma care. Recent LLMs extend beyond text processing and are now capable of analyzing, interpreting, and responding to user-uploaded medical images. Beginning with ChatGPT-4 Omni (GPT-4o), newer generations of LLMs have incorporated real-time multimodal capabilities, enabling the integration of textual, visual, and auditory inputs for complex diagnostic tasks (5–7). The recently released GPT-5 further enhances diagnostic reasoning, reduces hallucinations, and improves robustness across heterogeneous clinical inputs (5). Together, these advancements position LLMs as a promising frontier in medical artificial intelligence (AI) (8).

Compared with traditional dermatologic resources, LLMs offer distinct advantages, including continuous availability, low cost, and scalable diagnostic support. Consequently, the public increasingly uses them for medication information, self-diagnosis, and disease-prevention guidance (9–13). Clinicians and medical students also explore LLMs for knowledge acquisition and clinical decision support (11, 14, 15).

Numerous studies have investigated the use of LLMs for melanoma detection. Shifai et al. evaluated GPT-4 Vision (GPT-4 V) using 100 dermoscopic images randomly selected from the International Skin Imaging Collaboration (ISIC) Archive (16). Similarly, Liu et al. analyzed 100 ISIC images to compare GPT-4 V with another LLM, Claude 3 Opus (17). Sattler et al. assessed the performance of GPT-4 Turbo (GPT-4 T) and GPT-4o for melanoma identification using the Human Against Machine with 10,000 training images (HAM10K) dataset, reporting variable sensitivity, specificity, and accuracy (18). In another study, Boostani et al. compared ChatGPT-4o with Gemini 2.0 Flash and found that model performance differed between clinical and dermoscopic images; notably, the combined use of both models enabled identification of melanoma in >90% of cases (19). More recently, Wang et al. evaluated GPT-5 on images from both ISIC and HAM10K, demonstrating moderate performance in top-1 prediction and malignancy discrimination, along with substantially improved top-3 diagnostic accuracy (20).

In addition to the ISIC and HAM10K, other datasets such as PH^2^ have also been used to evaluate LLMs (21). However, these datasets predominantly contain images of light-skinned individuals and lack standardized skin tone annotations, limiting conclusions regarding model performance across all populations. Importantly, previous studies have shown that AI-assisted dermatologic diagnosis is less accurate for images of darker skin compared with lighter skin (22), underscoring the need for rigorous evaluation of LLMs across diverse cutaneous phenotypes to ensure safe and equitable applications. Prior attempts to examine variation in skin tone have been constrained by small sample sizes (23), and these assessments do not reflect the capabilities of newer models. To address this gap, we systematically evaluated the newly released GPT-5.2 across diverse skin complexions. To the best of our knowledge, this study represents the first systematic evaluation of GPT-5.2 for melanoma diagnosis across explicitly stratified skin tone groups using a dermatologic dataset with standardized skin tone annotations.

## 2 Methods

Despite rapid advances and growing interest in dermatologic AI, publicly available, expertly curated, and pathologically confirmed melanoma image datasets with diverse skin tones remain limited and difficult to access (24, 25). After surveying available dermoscopic image resources, we identified the Multimodal Imaging Learning Kit with 10,480 images (Milk10K) as the most suitable dataset for comparing LLM performance across skin tones (25). Milk10K is a clinically curated dermatology benchmark dataset that provides histopathology-confirmed diagnoses and multimodal imaging, thereby offering a reliable resource for evaluating diagnostic algorithms across diverse populations. The Milk10K dataset comprises 5,240 image pairs consisting of dermoscopic and corresponding clinical close-up images, each accompanied by diagnostic labels and skin tone annotations (25).

Skin pigmentation in Milk10K is annotated using an ordinal variable ranging from 0 (darkest) to 5 (lightest), inspired by the Fitzpatrick and Monk skin tone frameworks (26). For simplicity, we refer to this variable as MST (Milk10K Skin Tone) throughout this study.” No other changes are needed. Since the number of cases labeled as class 0 was very small (*n* = 6), this category was merged with class 1 prior to analysis. Milk10K additionally provides Fitzpatrick skin type annotations (I–VI) as auxiliary metadata; however, the MST-derived variable was used as the primary stratification variable in our analyses.

To enable a controlled evaluation of model performance across pigmentation groups, we constructed a balanced subset stratified by skin tone class. After merging MST classes 0 and 1, the combined MST 0/1 category contained 111 lesions. Lesions labeled as indeterminate epidermal proliferation represent diagnostically ambiguous entities that cannot reliably be categorized as benign or malignant and were therefore excluded from the analysis. Following this exclusion, the merged MST 0/1 group comprised 92 eligible lesions. We then randomly sampled 92 lesions from each of the remaining skin tone classes (MST 2–5) to achieve equal representation across groups. This stratified sampling strategy yielded a final evaluation cohort of 460 unique lesions (92 per skin tone group) and 920 corresponding images, which were used consistently across all experiments.

Given that major LLM-based benchmarks in this field have primarily evaluated ChatGPT models (16, 18, 20), this study focuses on GPT-5.2, a leading model released by OpenAI in December 2025, to facilitate comparability with prior analyses. Since previous studies have demonstrated that GPT-5 is not well-suited for top-1 diagnosis (20), defined as the accuracy of the model’s single highest-ranked prediction, we did not include top-1 evaluation in the present investigation. Instead, GPT-5.2 was evaluated across two related diagnostic tasks: (A) top-3 differential diagnosis, defined as the ordered list of the three diagnoses ranked most likely by the model, and (B) malignancy discrimination, a binary classification of lesions as malignant or benign. For the top-3 task, a prediction was considered correct if the ground-truth diagnosis appeared anywhere among the model’s three highest-ranked outputs.

GPT-5.2 was accessed programmatically through the OpenAI Application Programming Interface (API). To reflect real-world deployment, the model was used “as is,” without fine-tuning or external training. Dermoscopic images were submitted to GPT-5.2 with standardized prompts, which consisted of two components: (1) an instruction specifying the diagnostic task and (2) a formatting instruction requesting output in JSON format for standardized downstream analysis. Model responses were parsed, stored, and compared with ground-truth clinical diagnoses using a custom analysis pipeline implemented in Python (version 3.11.10). The Python scripts developed for this study are publicly available at https://github.com/qwangmsk/Melanoma-Detect.

Model performance was evaluated using sensitivity, specificity, accuracy, F1 score, and related metrics, which were computed using R (version 4.3.3) and visualized using the ggplot2 package (version 3.5.1). To assess potential differences across skin tone groups, pairwise comparisons were performed using bootstrap resampling distributions. Two-sided *p*-values were derived from the empirical distributions of F1 score differences and were adjusted using the Benjamini–Hochberg procedure to control the false discovery rate.

## 3 Results

Using the evaluation cohort described in the Methods, GPT-5.2 predictions were generated for each lesion under both imaging conditions (dermoscopy-only and dermoscopy plus clinical close-up images) and across the two diagnostic tasks (top-3 differential diagnosis and binary malignancy discrimination). Model outputs were standardized and compared with ground-truth labels to compute performance metrics, both overall and stratified by skin tone class. These results were used to generate the summary tables and figures presented below, as well as Supplementary Tables S1–S3 and Supplementary Figure S1.

**Figure 1:**
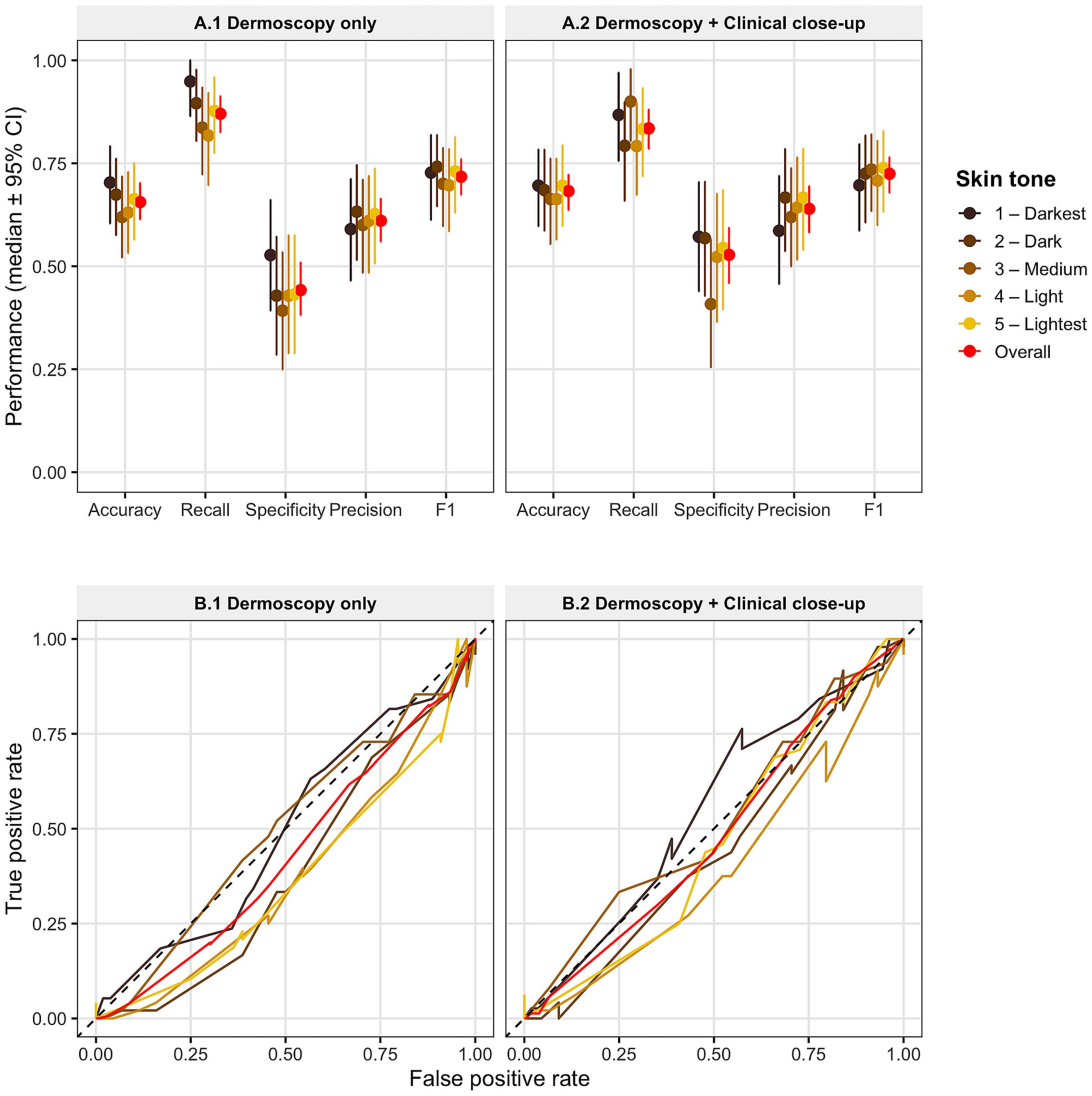
GPT-5.2 top-3 differential diagnosis performance across skin tone groups. **(A)** Top-3 diagnostic performance metrics across skin tone classes for two input conditions: dermoscopy-only **(A1)** and dermoscopy plus paired clinical close-up images **(A2).** A prediction was considered correct if the ground-truth diagnosis appeared among the model’s three highest-ranked outputs. Points represent median values, and error bars indicate 95% confidence intervals for accuracy, recall, specificity, precision, and F1 score. Colored markers denote individual skin tone classes, with darker colors corresponding to darker skin tones; red markers indicate overall performance across all tones. **(B)** Receiver operating characteristic (ROC) curves for the same two input conditions: dermoscopy-only **(B1)** and dermoscopy plus clinical close-up conditions **(B2)**, shown separately for each skin tone class and for the overall dataset. ROC curves were generated using the confidence score associated with the model’s highest-ranked prediction for each lesion; therefore, they reflect discrimination performance for the top-1 diagnosis rather than the full top-3 differential list. Consistent with prior observations, top-1 diagnostic performance is substantially lower than performance based on the full top-3 differential outputs, as shown in **(A1,A2)**. The dashed diagonal line represents the reference line for random classification. Analyses were conducted on a balanced subset of 460 lesions from the Milk10K dataset (92 lesions per skin tone group), with paired dermoscopic and clinical close-up images available for each lesion.

Table 1 and Figure 1 summarize GPT-5.2 performance for the top-3 differential diagnosis task across skin tone classes under both dermoscopy-only and combined dermoscopy plus clinical close-up settings. Across all evaluated metrics, including accuracy, recall, specificity, precision, F1 score, and Cohen’s kappa (*κ*), performance was broadly consistent across pigmentation groups, with no systematic decline observed in darker skin tones compared to lighter skin tones. Although modest variability was present among individual skin tone classes, the performance estimates overlapped substantially, and confidence intervals (CIs) exhibited considerable concordance, indicating the absence of a clear skin tone-dependent performance gradient. These findings suggest that GPT-5.2 prioritizes diagnostically relevant candidates with comparable effectiveness across diverse pigmentation levels.

**Table 1:** GPT-5.2 top-3 differential diagnosis performance across skin tones.

(a) Dermoscopy only							
Skin tone class	*N*	Accuracy	Recall	Specificity	Precision	F1 score	Kappa
1	92	0.703	0.947	0.528	0.590	0.727	0.438
2	92	0.674	0.896	0.432	0.632	0.741	0.334
3	92	0.620	0.833	0.386	0.597	0.696	0.224
4	92	0.630	0.813	0.432	0.609	0.696	0.248
5	92	0.663	0.875	0.432	0.627	0.730	0.312
Overall	460	0.658	0.870	0.445	0.612	0.718	0.315

(b) Dermoscopy + clinical close-up							
Skin tone class	*N*	Accuracy	Recall	Specificity	Precision	F1 score	Kappa
1	92	0.696	0.868	0.574	0.589	0.702	0.413
2	92	0.685	0.792	0.568	0.667	0.724	0.363
3	92	0.663	0.896	0.409	0.623	0.735	0.311
4	92	0.663	0.792	0.523	0.644	0.710	0.318
5	92	0.696	0.833	0.545	0.667	0.741	0.383
Overall	460	0.680	0.835	0.526	0.638	0.723	0.361

The addition of clinical close-up images resulted in modest overall improvements compared with dermoscopy alone (Table 1), particularly for specificity, precision, and Cohen’s *κ*, while recall remained relatively stable across skin tones. Importantly, these improvements were observed across all pigmentation groups without evidence of differential benefit by skin tone. Overall, GPT-5.2 exhibited stable performance for top-3 differential diagnoses across diverse skin tones on the Milk10K dataset, with no statistically significant disparities attributable to pigmentation level.

As shown in Figures 1B1,B2, the receiver operating characteristic (ROC) analyses were constructed using the confidence score associated with the model’s highest-ranked prediction for each lesion; therefore, they primarily reflect discrimination performance for the top-1 diagnosis rather than the full top-3 differential list. Notably, a recent study by Wang et al. also reported relatively lower performance for GPT-5 when evaluated using only the top-1 diagnosis (20), which aligns with the ROC patterns observed for GPT-5.2 in the present study. The consistent pattern demonstrated in both the present study and Wang et al.’s study, in which top-1 diagnostic performance is lower than top-3 performance, provides additional validation that model accuracy improves when multiple differential diagnoses are considered.

**Figure 2:**
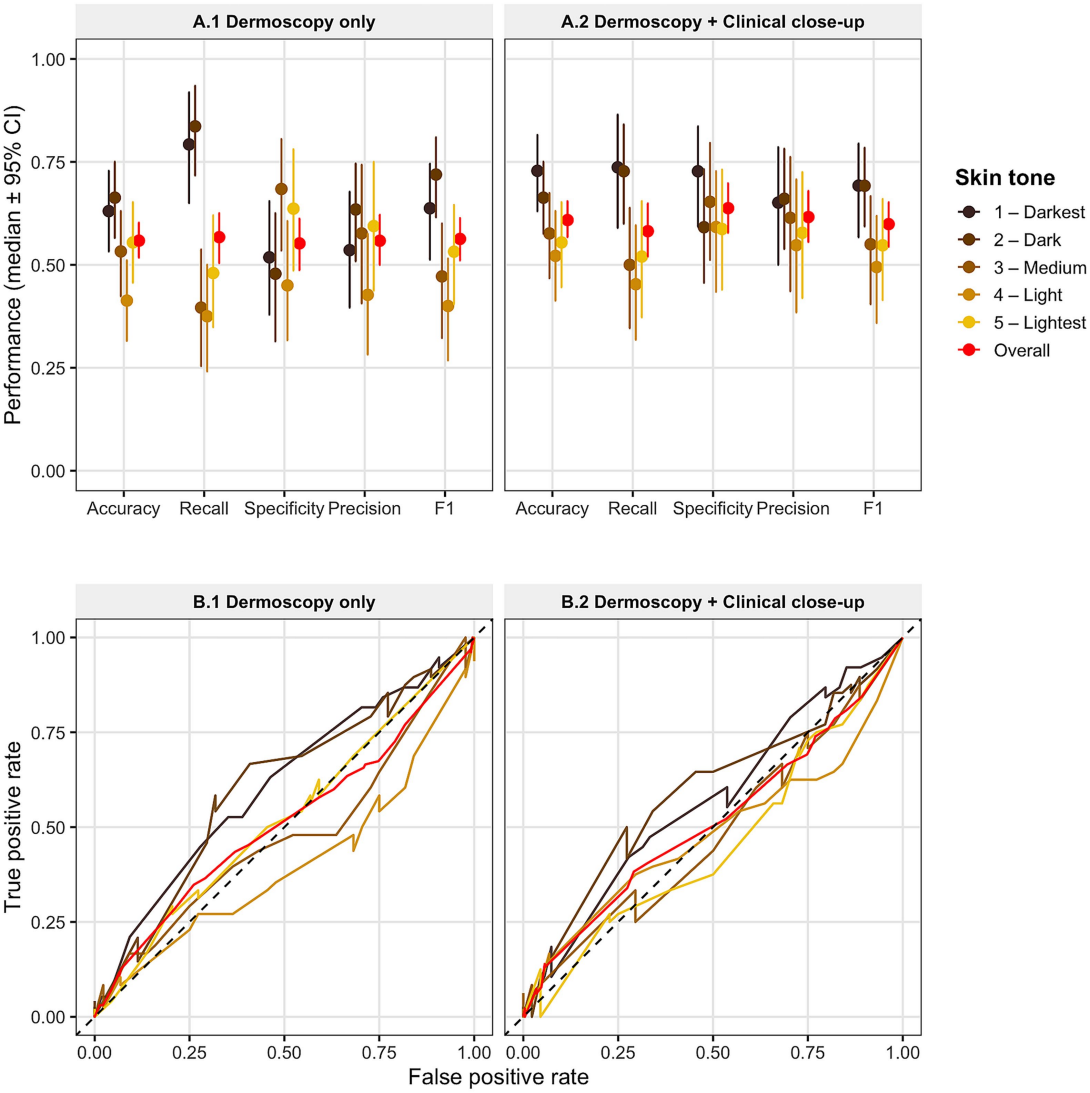
GPT-5.2 melanoma discrimination performance across skin tone groups. **(A)** Performance metrics across skin tone classes for two input conditions: dermoscopy-only **(A1)** and dermoscopy plus paired clinical close-up images **(A2)**. Points represent median values, and error bars indicate 95% confidence intervals for accuracy, recall, specificity, precision, and F1 score. Colored markers denote individual skin tone classes, with darker colors corresponding to darker skin tones; red markers indicate overall performance across all tones. **(B)** ROC curves for the same two input conditions: dermoscopy-only **(B1)** and dermoscopy plus clinical close-up conditions **(B2)**. Curves are shown separately for each skin tone class and for the overall dataset. The dashed diagonal line represents the reference line for random classification. Analyses were conducted on a balanced subset of 460 lesions from the Milk10K dataset (92 lesions per skin tone group), with paired dermoscopic and clinical close-up images available for each lesion. Confidence scores produced by GPT-5.2 were used to generate ROC curves.

Consistent with the findings for differential diagnosis prioritization, GPT-5.2 showed broadly comparable performance across skin tone classes in the binary malignancy discrimination task under both dermoscopy-only and combined dermoscopy plus clinical close-up conditions (Table 2; Figure 2). Across all evaluated metrics, no consistent pattern of performance decline was observed from lighter to darker skin tones. Although some variability was present among individual skin tone groups, pairwise comparisons based on bootstrap-derived F1 score distributions did not reveal statistically significant differences after multiple comparison adjustment. These findings are consistent with the substantial overlap in confidence intervals observed across skin tone groups, as shown in Figure 2.

**Table 2:** GPT-5.2 malignancy discrimination performance across skin tones.

(a) Dermoscopy only							
Skin tone class	*N*	Accuracy	Recall	Specificity	Precision	F1 score	Kappa
1	92	0.630	0.789	0.519	0.536	0.638	0.288
2	92	0.663	0.833	0.477	0.635	0.721	0.315
3	92	0.533	0.396	0.682	0.576	0.469	0.077
4	92	0.413	0.375	0.455	0.429	0.400	−0.169
5	92	0.554	0.479	0.636	0.590	0.529	0.115
Overall	460	0.559	0.565	0.552	0.558	0.562	0.117

(b) Dermoscopy + clinical close-up							
Skin tone class	*N*	Accuracy	Recall	Specificity	Precision	F1 score	Kappa
1	92	0.728	0.737	0.722	0.651	0.691	0.450
2	92	0.663	0.729	0.591	0.660	0.693	0.322
3	92	0.576	0.500	0.659	0.615	0.552	0.158
4	92	0.522	0.458	0.591	0.550	0.500	0.049
5	92	0.554	0.521	0.591	0.581	0.549	0.111
Overall	460	0.609	0.583	0.635	0.615	0.598	0.217

The incorporation of paired clinical close-up images resulted in modest improvements in overall performance metrics; however, these gains were observed across multiple skin tone groups rather than being confined to any specific pigmentation category. ROC curves similarly demonstrated comparable discrimination ability across skin tones in both imaging scenarios (Figure 2). Collectively, these findings suggest that GPT-5.2’s malignancy discrimination performance on the Milk10K dataset is largely consistent across diverse skin pigmentation levels, with no evidence of significant performance disparity between darker and lighter skin tones. Taken together with the top-3 diagnosis results, the overall evidence suggests that GPT-5.2 maintains consistency in melanoma-related diagnostic performance across skin tone classes on this dataset. To provide additional clinical context for the malignancy discrimination task, Supplementary Table S3 compares GPT-5.2 with published clinician benchmarks for melanoma diagnosis. These contextual comparisons indicate that GPT-5.2 remains below the performance range reported for dermatologists.

Compared with the prior study evaluating GPT-5 on the dermoscopic datasets ISIC Archive and HAM10K (20), GPT-5.2 demonstrated comparable but generally slightly lower performance on the Milk10K dataset (see Supplementary Table S1; Supplementary Figure S1 for direct comparison). In Wang et al.’s study (20), GPT-5 achieved higher diagnostic metrics, whereas in the present study, GPT-5.2 reached top-3 diagnostic accuracy of approximately 0.66–0.68 with F1 scores of approximately 0.72, and malignancy discrimination accuracy of 0.56–0.61 with F1 scores of 0.56–0.60. These differences likely reflect the greater clinical heterogeneity and multimodal complexity of Milk10K compared with the ISIC Archive and HAM10K, rather than intrinsic model limitations. Importantly, a consistent pattern was observed in both the present study and Wang et al.’s study, in which GPT’s top-3 diagnostic performance exceeds malignancy discrimination performance, further supporting the validity of the present findings across datasets and model versions.

## 4 Discussion

To the best of our knowledge, this study represents one of the first systematic evaluations of a GPT-5.2 model across explicitly stratified skin tone groups using a large, pathologically confirmed dermatology dataset. Prior benchmarking studies of LLMs for melanoma detection have primarily relied on private datasets or public resources such as ISIC or HAM10K, which contain predominantly lighter skin types and lack standardized pigmentation annotations, thereby limiting conclusions regarding algorithmic equity. By leveraging the Milk10K dataset with skin tone-derived labels and balanced sampling across pigmentation classes, our study directly addresses this critical gap. Importantly, the absence of statistically meaningful performance differences across skin tones suggests that GPT-5.2 does not exhibit systematic degradation in diagnostic performance in darker skin within this dataset, in contrast to many AI-assisted systems that underperform in darker skin populations.

While GPT-5.2 exhibited moderate overall diagnostic accuracy, the stability of performance across pigmentation groups indicates that recent advances in LLM architecture may help mitigate some of the sources of skin tone-related bias reported in earlier computer vision systems. However, the performance variability across individual lesions and the only moderate agreement with ground truth underscore that the model is not suited to autonomous diagnosis. Accordingly, these findings should be interpreted as evidence of relative equity in model behavior rather than clinical readiness.

Several limitations of this study should be noted. First, although Milk10K provides explicit skin tone annotations and multimodal imaging, the dataset remains a curated research resource and may not fully capture the complexity of real-world clinical presentations. Consequently, the generalizability of these findings to routine clinical settings remains unclear. Second, the evaluation was performed using a balanced subset of lesions stratified by skin tone. This sampling strategy was intentionally adopted to minimize confounding due to unequal skin tone representation in the original dataset. However, since the resulting cohort does not reflect the true prevalence of melanoma in clinical populations, the reported performance metrics should be interpreted as comparative estimates under controlled conditions rather than direct estimates of clinical screening performance. Third, model performance was assessed using static images without additional clinical context (e.g., patient history, lesion evolution, or dermoscopic metadata) that clinicians typically incorporate into diagnostic reasoning, and the evaluation was conducted retrospectively on a single dataset due to the limited availability of public datasets with skin tone annotations. To support transparency and external validation, the identifiers of the 460 lesions included in this evaluation have been publicly released through our GitHub repository, enabling other investigators to reproduce our analysis or benchmark alternative models on the same cases. Finally, we did not assess calibration, decision thresholds, or user interaction effects, all of which are important considerations for clinical deployment. Future studies incorporating prospective validation, diverse patient populations, and human–AI interaction paradigms are needed to fully characterize the safety, reliability, and equity of LLM-based dermatologic decision support tools.

Given its moderate diagnostic performance and variability across cases, GPT-5.2 is best positioned as a clinician-supervised decision support tool rather than a standalone diagnostic system. Potential use cases include triage support, where the model may assist in identifying lesions that warrant expedited specialist evaluation, and educational support for trainees or patients seeking preliminary information. Importantly, the risk of false-negative predictions underscores the need for conservative triage strategies and clinician verification, particularly for lesions with ambiguous or high-risk features. In addition, practical considerations such as user interaction design, patient engagement, and medico-legal responsibility must be carefully addressed before clinical deployment. Accordingly, such systems should be considered adjunctive tools that complement, rather than replace, clinician expertise.

## Data Availability

Publicly available datasets were analyzed in this study. This data can be found at: https://api.isic-archive.com/doi/milk10k/.
