# Supplementary material for: Evaluation of GPT-5.2 for melanoma detection across skin tones
Source: Front Med (Lausanne). 2026 May 8;13:1816102. doi: 10.3389/fmed.2026.1816102 (PMC13194033; doi:10.3389/fmed.2026.1816102)
Supplement: Supplementary file 1 [file Data_sheet_1.docx]

Supplementary Material

# Supplementary Tables and Figures

## Comparison of our assessment of GPT-5.2 with a previous benchmark study of GPT-5

A recent benchmark study by Wang et al. systematically evaluated GPT-5 performance in melanoma detection using 100 and 500 randomly selected dermoscopic images from two public benchmark datasets, the ISIC Archive (<https://api.isic-archive.com/images/>) and HAM10K (<https://www.kaggle.com/datasets/kmader/skin-cancer-mnist-ham10000>), respectively (1). Because GPT-5 demonstrated stronger diagnostic performance on HAM10K than on the ISIC Archive, our analysis here focuses on comparing the performance of GPT-5.2 with the previously reported results obtained on HAM10K. Notably, the HAM10K dataset was derived largely from European clinical cohorts and predominantly contains images from individuals with lighter skin tones.

**Table S1** and **Figure S1** present our evaluation of GPT-5.2 on the Milk10K dataset together with the previously reported GPT-5 results on HAM10K (1). For the top-3 differential diagnosis task, GPT-5.2 achieved lower overall performance on Milk10K than GPT-5 achieved on HAM10K. In contrast, for the binary malignancy discrimination task, GPT-5.2 demonstrated broadly comparable accuracy and F1 scores when paired clinical close-up images were incorporated.

The apparent discrepancy between the two benchmark studies likely reflects differences in dataset composition rather than a decline in model capability. Compared with HAM10K, the Milk10K dataset contains a broader spectrum of diagnostic categories, includes both dermoscopic and clinical images, and incorporates more heterogeneous real-world imaging conditions and greater representation of diverse skin tones. These factors substantially increase the complexity of the diagnostic task and therefore represent a more challenging benchmark for automated diagnostic systems, including multimodal large language models such as GPT-5.2.

**Table S1.** Comparison of the diagnosis performance of GPT-5.2 on the Milk10K dataset with that of GPT-5 on the HAM10K dataset

| ****Model (Scenario)**** | ****Diagnostic Objective**** | Recall ****(%)**** | ****Specificity (%)**** | ****Accuracy (%)**** | ****F1 (%)**** | ****FNR**^#^ **(%)**** | ****Kappa**** |
| --- | --- | --- | --- | --- | --- | --- | --- |
| GPT-5 (dermoscopy) ^+^ | Top 3 **diagnosis** | **100** | **86.2** | 92.0 | 91.3 | 6.8 |  |
|  | Malignancy discrimination | **56.0** | **72.0** | 64.0 | **60.9** | **34.9** | **0.417** |
| GPT-5.2 (dermoscopy) | Top 3 **diagnosis** | **87.0** | **44.5** | 65.8 | 71.8 | 13.0 |  |
|  | Malignancy discrimination | **58.3** | **56.6** | 57.4 | 54.8 | 43.3 | 0.148 |
| GPT-5.2 (dermoscopy ^#^ closeup) | Top 3 **diagnosis** | **83.5** | **52.6** | 68.0 | 72.3 | 16.5 |  |
|  | Malignancy discrimination | **66.7** | **62.6** | 64.3 | 61.7 | 43.5 | 0.287 |

^+^ **GPT-5** performance on the HAM10K dataset was extracted from a published paper (except for FNR) (1). Notably, HAM10K contains only dermoscopic images and does not include corresponding clinical close-up photographs.

**^#^ FNR**: false negative rate. The FNR of GPT-5 was calculated from the confusion matrices published by Wang et al. in paper (1).


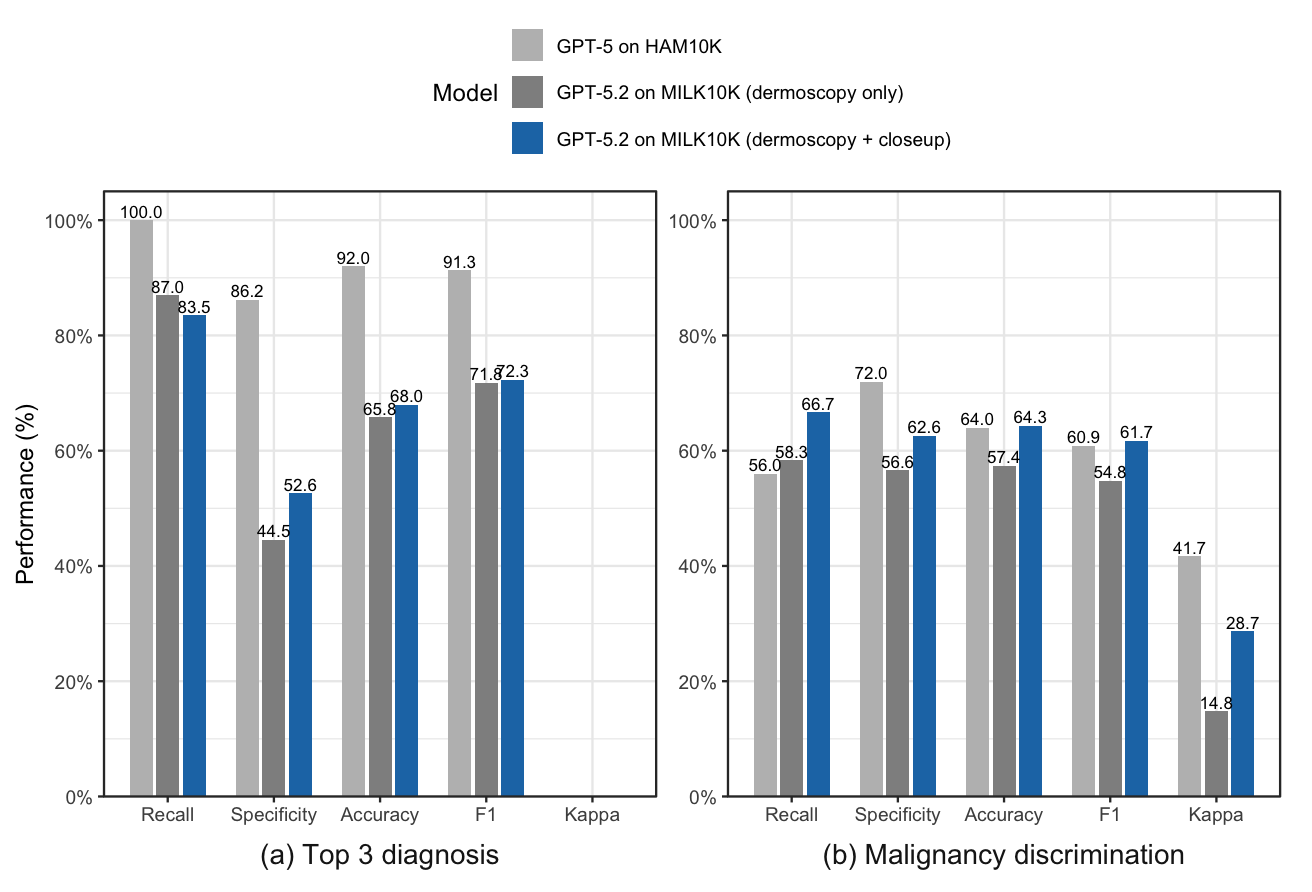


**Figure S1. Comparison of GPT-5 and GPT-5.2 performance across datasets.** Performance of GPT-5 on the HAM10K dataset and GPT-5.2 on the Milk10K dataset for (a) top-3 differential diagnosis and (b) malignancy discrimination (melanoma vs. benign). Data are derived from Table S1.

## Comparison of GPT-5.2 with a deep CNN model, ResNet-50, for differential diagnoses of melanoma on Milk10K

The Milk10K developers provided a baseline machine-learning pipeline based on ResNet-50, a 50-layer convolutional neural network (CNN) from the residual learning family, to demonstrate multiclass classification using paired dermoscopic and clinical close-up images (2). This section compares our assessment of GPT-5.2 with the differential diagnosis results reported for this ResNet-50 model (2). The differential diagnosis of the models was considered correct if the ground-truth diagnosis appeared among the model’s three highest-ranked outputs.

**Table S2** summarizes the comparative performance of GPT-5.2 and ResNet-50 on the Milk10K dataset using paired dermoscopic images and clinical close-up photographs as input. GPT-5.2 exhibited substantially higher recall/sensitivity for identifying the correct diagnosis within its top-3 predictions, whereas ResNet-50 achieved markedly higher specificity, reflecting a more conservative prediction strategy. Despite this trade-off, the two models' overall accuracy was comparable (0.680 vs. 0.677).

| **Model** | **Recall (95% CI)** ^+^ | **Specificity (95% CI)** | **Accuracy (95% CI)** | **Notes** |
| --- | --- | --- | --- | --- |
| GPT-5.2 | 0.835 (0.786 - 0.880) | 0.526 (0.460 - 0.593) | 0.680 (0.637-0.722) | Present study |
| ResNet-50 ^#^ | 0.426 (0.392 - 0.459) | 0.960 (0.959 - 0.961) | 0.677 (0.662–0.692) | Published result |

**Table S2.** Top-3 differential diagnosis performance of GPT 5.2 and ResNet-50 on Milk10K

^+^ **CI:** Confidence Interval

^#^ **ResNet-50** performance was extracted from the published MILK10k paper (2). The training and testing of the model on MILK10k is described in detail at <https://codeberg.org/ptschandl/MILK10k_train_base>.

## Comparison of GPT-5.2 malignancy discrimination with published clinician benchmarks for melanoma

To provide additional clinical context, we compared the performance of GPT-5.2 on the Milk10K dataset with published physician benchmarks from a recent systematic review and meta-analysis of melanoma diagnostic accuracy for the binary malignancy discrimination (3). That study reported pooled sensitivity and specificity estimates for melanoma diagnosis stratified by evaluator type and examination method, including experienced dermatologists, inexperienced dermatologists, and primary care physicians using either in-person clinical examination/images or dermoscopy/dermoscopic images. Specifically, for melanoma diagnosis using dermoscopy/dermoscopic images, reported sensitivity and specificity were 85.7% and 81.3%, respectively, for experienced dermatologists; 78.0% and 69.5% for inexperienced dermatologists; and 49.5% and 91.3% for primary care physicians (3).

**Table S3** places these published clinician estimates alongside our GPT-5.2 results. On Milk10K, GPT-5.2 achieved malignancy-discrimination sensitivity of 56.5% using dermoscopy alone and 58.3% when paired clinical close-up images were incorporated, with corresponding specificity of 55.2% and 63.5%, respectively. These findings indicate that GPT-5.2 remains below the performance range reported for dermatologists and below the specificity reported for primary care physicians in melanoma detection.

This comparison is intended to provide clinical context rather than a direct head-to-head benchmark. The published clinician estimates were derived from heterogeneous studies pooled in a meta-analysis, whereas GPT-5.2 was evaluated retrospectively on a research dataset with balanced skin-tone sampling. In addition, the published study synthesized results across different evaluators, clinical settings, and examination methods, whereas the present analysis used standardized model prompts and image inputs on the Milk10K benchmark. These methodological differences limit direct comparability but may help frame the observed GPT-5.2 performance relative to clinically reported melanoma diagnostic accuracy.

**Table S3**. Contextual comparison of GPT-5.2 malignancy-discrimination performance on Milk10K with published clinician benchmarks for melanoma diagnosis. Published clinician estimates were extracted from a review article and are provided for context rather than head-to-head comparison.

| **Evaluator** ^+^ | **Method** | **Sensitivity %**  **(95% CI)** ^#^ | **Specificity %**  **(95% CI)** |
| --- | --- | --- | --- |
| Experienced dermatologist | Clinical examination/images | 76.9 (69.3–83.1) | 89.1 (76.9–95.3) |
| Experienced dermatologist | Dermoscopy/images | 85.7 (82.5–88.3) | 81.3 (76.3–85.4) |
| Inexperienced dermatologist | Clinical images | 78.3 (54.9–91.4) | 66.2 (55.9–75.1) |
| Inexperienced dermatologist | Dermoscopic images | 78.0 (69.3–84.7) | 69.5 (52.9–82.2) |
| Primary care physician | Clinical examination | 37.5 (21.1–56.3) | 84.6 (80.0–88.5) |
| Primary care physician | Dermoscopy/images | 49.5 (40.4–58.6) | 91.3 (78.0–96.9) |
| GPT-5.2 | Dermoscopy only | 56.5 (50.4-62.5) | 55.2 (48.7-61.2) |
| GPT-5.2 | Dermoscopy+clinical close-up | 58.3 (52.1-64.9) | 63.5 (57.8-69.8) |

^+^ **Note:** Published physician values were extracted from Chen et al., *JAMA Dermatology* 2025, Table 2 (3).

^#^ **CI:** Confidence Interval

# References

1. Wang Q, Amugo I, Rajakaruna H, Irudayam MJ, Xie H, Shanker A, et al. Evaluating GPT-5 for Melanoma Detection Using Dermoscopic Images. Diagnostics. 2025;15(23):3052.

2. Tschandl P, Akay BN, Rosendahl C, Rotemberg V, Todorovska V, Weber J, et al. MILK10k: A Hierarchical Multimodal Imaging-Learning Toolkit for Diagnosing Pigmented and Nonpigmented Skin Cancer and its Simulators. Journal of Investigative Dermatology. 2025.

3. Chen JY, Fernandez K, Fadadu RP, Reddy R, Kim M-O, Tan J, et al. Skin Cancer Diagnosis by Lesion, Physician, and Examination Type. JAMA Dermatology. 2025;161(2):135.
